# Technology in Exercise Interventions for Older Women: Acceptability, Adherence, and Special Considerations

**DOI:** 10.1093/geroni/igaa057.3091

**Published:** 2020-12-16

**Authors:** Shannon Halloway

**Affiliations:** Rush University, Chicago, Illinois, United States

## Abstract

Women aged 65 years and older participate in less moderate-vigorous physical activity (PA) than men of this age group, which increases the risk for a myriad of chronic health problems. Interventions that utilize a lifestyle approach to increase PA in everyday life are preferred by women compared to structured exercise, but long-term adherence is a challenge. Technology (e.g., wearables, social media, computers) can be efficiently leveraged as motivational tools in lifestyle PA interventions. However, the unique needs of older women must be considered. Thus, the purpose is to examine the: (a) types of technology that were successfully integrated into existing PA intervention studies designed for older women; (b) acceptability and adherence to technological approaches by older women; and (c) additional considerations needed for special populations, including older women with chronic health problems. The efficiency and scalability of technological approaches with clinical and public health implications will also be discussed.

